# Aicardi-Goutières syndrome type 6: report of *ADAR* variant and clinical outcome after ruxolitinib treatment in the neonatal period

**DOI:** 10.1186/s12969-024-01036-5

**Published:** 2024-12-28

**Authors:** Alba Gabaldon-Albero, Carla Martin-Grau, Miguel Marti-Masanet, Alejandro Lopez-Jimenez, Roberto Llorens, Beatriz Beseler-Soto, Sergio Martin-Zamora, Berta Lopez, Inmaculada Calvo, Sara Hernandez-Muela, Monica Rosello, Carmen Orellana, Francisco Martinez

**Affiliations:** 1 Pediatric Neurology Unit, Hospital Universitario y Politécnico La Fe, Valencia, Spain; 2 Translational Genetics Research Group, La Fe Health Research Institute (IIS La Fe), Avenida Fernando Abril Martorell nº 106 Tower A, 7th Floor, Valencia, Spain; 3 Present Address: Genetics Unit, Hospital Universitario y Politécnico La Fe, Avenida Fernando Abril Martorell nº 106 Tower A, 4th Floor, Valencia, Spain; 4 Pediatric Rheumatology Unit, Hospital Universitario y Politécnico La Fe, Valencia, Spain; 5 Neonatology Unit, Hospital Universitario y Politécnico La Fe, Valencia, Spain; 6 Pediatric Radiology, Unit Hospital Universitario y Politécnico La Fe, Valencia, Spain

**Keywords:** Aicardi-goutières syndrome, Type 1 interferonopathy, *ADAR* gene, Ruxolitinib, Infantile encephalopathy

## Abstract

**Background:**

Aicardi-Goutières Syndrome is a monogenic type 1 interferonopathy with infantile onset, characterized by a variable degree of neurological damage. Approximately 7% of Aicardi-Goutières Syndrome cases are caused by pathogenic variants in the *ADAR* gene and are classified as Aicardi-Goutières Syndrome type 6. Here, we present a new homozygous pathogenic variant in the *ADAR* gene. Currently, Janus Kinase inhibitors have been proposed to treat selected interferonopathies such as Aicardi-Goutières Syndrome, although limited information is available on its use and results in the neonatal presentation of this disease.

**Case presentation:**

We present two siblings, a male neonate with congenital petechial rash, severe thrombopenia and generalized hypotonia and his deceased sister who had normal development until 5 months of age, when she suffered acute encephalopathy. We describe the clinical course, complementary examinations and follow-up with early treatment of the newborn with ruxolitinib. The homozygous variant c.2908G > A (p.Ala970Thr) in the *ADAR* gene was found in both siblings, parents were heterozygous carriers.

**Conclusions:**

The homozygous variant c.2908G > A (p.Ala970Thr) in the *ADAR* gene causes Aicardi-Goutières Syndrome type 6. Intrafamilial phenotypic spectrum of the disease varies among individuals with the same pathogenic variant. Early initiation of ruxolitinib improved systemic signs but did not prevent the progression of neurological disease.

## Background

Aicardi-Goutières Syndrome (AGS) is a monogenic type 1 interferonopathy with onset in infancy. AGS manifests as an inflammatory early-onset disease, which main disorder is an encephalopathy resulting, in most cases, in severe neurological damage. It is characterized by intracranial calcification, white matter disease and cerebrospinal fluid (CSF) lymphocytosis. Additionally, due to upregulated interferon production, other systemic clinical features such as hepatosplenomegaly, elevated liver enzymes, fever of unknown origin and thrombocytopenia may be present in the neonatal period in 20% of cases, resembling a congenital infection [1].

To date, pathogenic variants in 9 genes (*ADAR*,* IFIH1*,* LSM11*,* RNASEH2A*,* RNASEH2B*,* RNASEH2C*,* RNU7-1*,* SAMHD1*,* TREX1*) cause AGS subtypes 1–9 with different inheritance patterns. AGS-6 (OMIM#615010) is due to homozygous or compound heterozygous pathogenic variants in *ADAR* gene, which accounts for 7% of AGS cases [1, 2].

ADAR (Adenosine Deaminase Acting on RNA) proteins are editing enzymes that mark dsRNA as self and suppress the type I interferon (IFN) response, acting as a form of negative feedback mechanism of inflammatory regulation. Thus, variants in *ADAR* are often associated with diseases involving upregulated IFN, which inflammatory action is mediated by the activation of two of the four human Janus Kinases. For this reason, Janus Kinase inhibitors (JAKIs) have been proposed to treat selected interferonopathies [2–5].

At present, more than 80 likely pathogenic/pathogenic variants in *ADAR* gene causative of AGS type 6 have been reported, most of them are missense variants [6, 7]. In this study, we report two siblings with AGS-6 caused by the homozygous variant c.2908G > A (p.Ala970Thr) in the *ADAR* gene, their different clinical course and the response to ruxolitinib in one of them.

## Case presentation

**Fig. 1:**
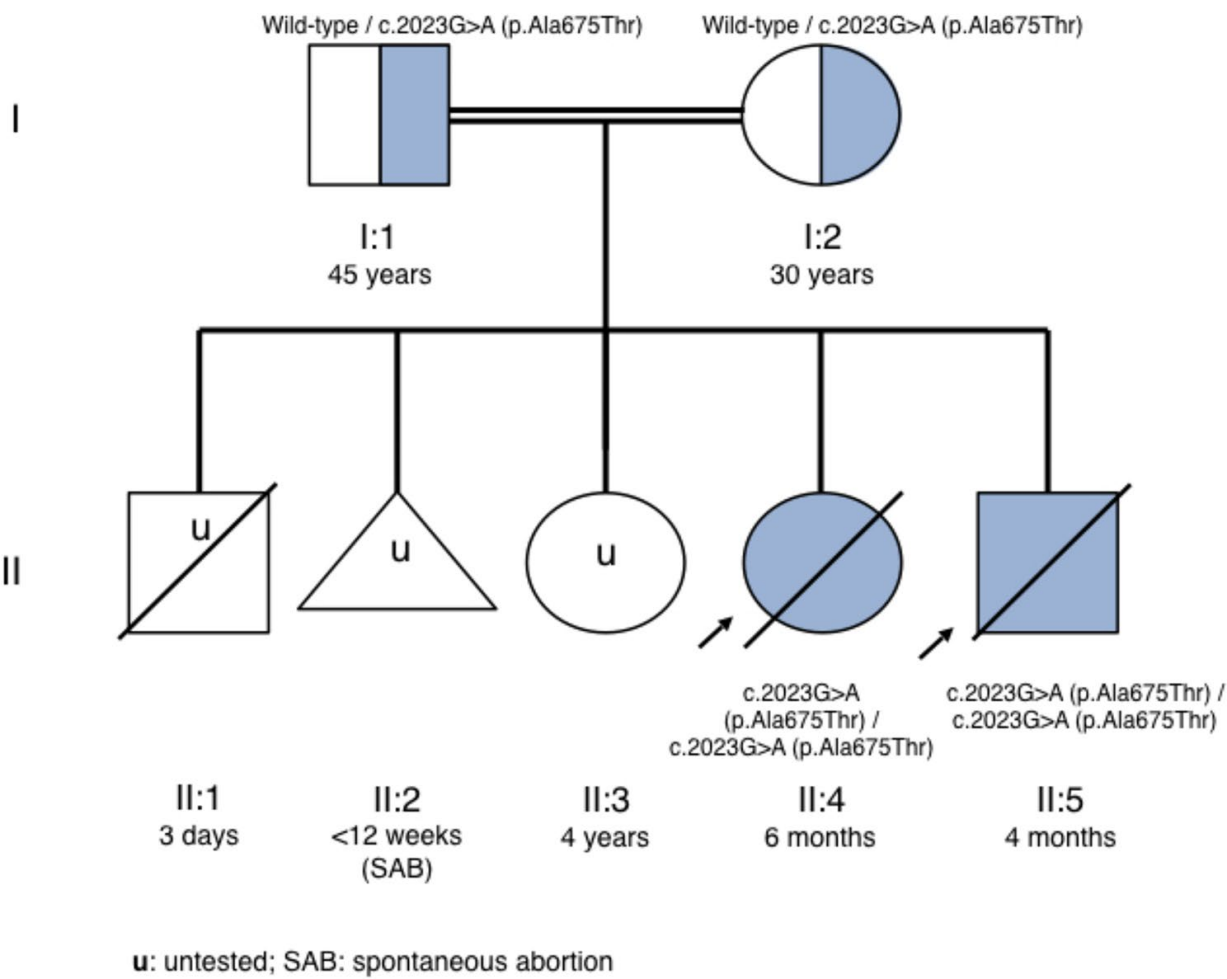
Pedigree of the reported family. The probands (II:4 and II:5) are indicated with an arrow. Heterozygous and homozygous carriers for the *ADAR* c.2908G > A; p.Ala970Thr (NM_001111.5) missense variant are shown in blue color

**Fig. 2:**
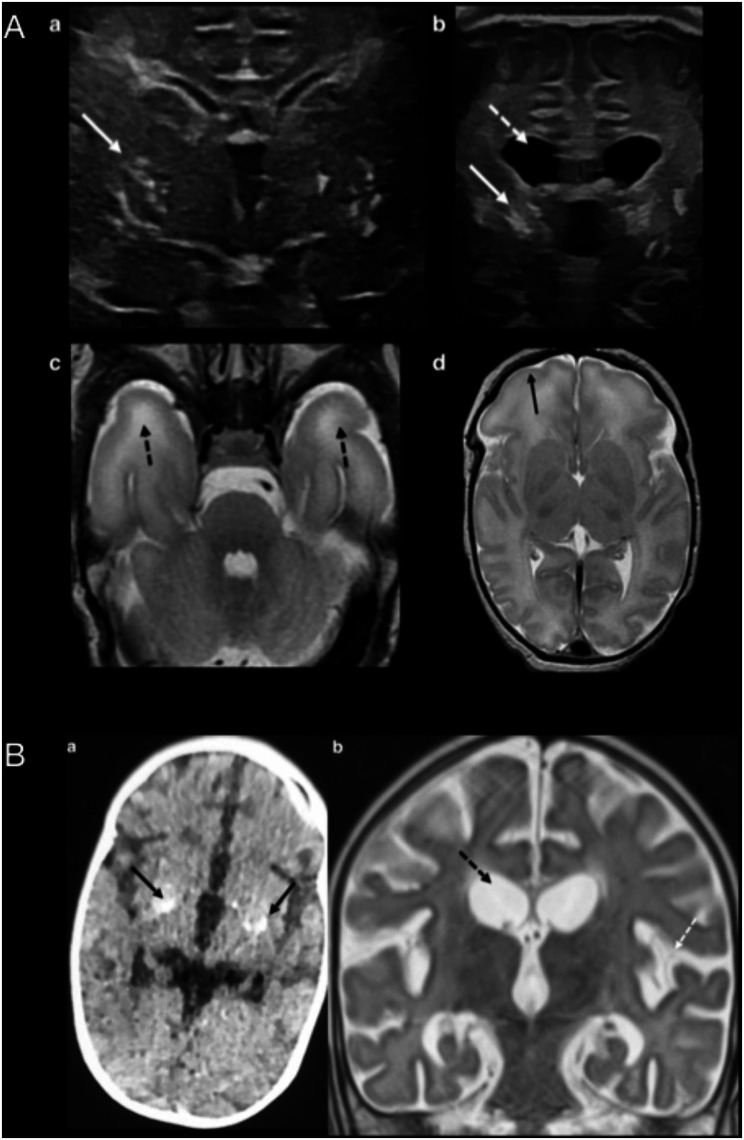
Neuroimaging findings. **A** Brain ultrasound (II:5) at birth (a) and at 3 months (b). Brain MRI T2-weighted axial images at 10 days (c and d). In (a) bilateral thalamostriate vasculopathy (white arrow) is shown. At 3 months (b) the extent of vasculopathy had increased (white arrow) and ventriculomegaly had developed (dashed white arrow). MRI (c) identified bilateral anterior temporal lobe white matter hyperintensity (dashed black arrows) and (d) simplified frontal lobe gyration pattern (black arrow). **B** Brain CT axial image (a) and coronal brain MRI T2-weighted (b) at 6-month-old (II:4). In (a) bilateral basal ganglia calcification is noticed (black arrows). In MRI (b) ventriculomegaly (dashed black arrow) and increased subarachnoid space (dashed white arrow) is remarkable

It was a consanguineous Moroccan family who were in their fifth pregnancy at the moment of admission in our hospital (Fig. 1). Concerning the background, the first pregnancy was a male (II:1) who was born in another center by spontaneous vaginal delivery at 32 weeks, was small for gestational age and died on his third day of life. More information on the cause of death is unknown. No genetic studies were performed. The second (II:2) was a spontaneous abortion before 12 weeks of pregnancy. The third is a healthy female until now (II:3). The fourth, is a deceased female (II:4) who was born at 36 weeks small for gestational age in our center. She had normal development until 5 months of age, when she suffered acute encephalopathy with absence of head support, inability to feed and continuous irritability. At 6 months, she was admitted in a Moroccan center, where the development of refractory status epilepticus is reported and caused her death. Her neuroimaging studies are described in Fig. 2B. A confirmatory diagnosis was not reached in this case. In the course of the fifth gestation, the parents requested an extension of the study of their previously deceased daughter (II.4). As a dried blood spot sample from the neonatal screening was available in our hospital, genetic study was processed. Genomic DNA of the patient (II:4) was extracted and clinical exome sequencing was performed using SureSelectXT HS Human All Exon kit V6 35.1 Mb (*Agilent Technologies*, CA, USA) for library preparation and target enrichment. Libraries were sequenced on Illumina NextSeq500 system (*Illumina*, CA, USA). Variant analysis, interpretation and family segregation study were performed as reported elsewhere [10]. The patient (II:4) was homozygous carrier of the likely pathogenic variant c.2908G > A (p.Ala970Thr) in *ADAR* gene and both parents were heterozygous carriers (Fig. 1).

The genetic diagnosis was obtained before the birth of the fifth child (II:5), however the parents declined prenatal diagnosis. The patient (II:5) was a male newborn with petechial rash, severe thrombopenia and generalized hypotonia. During pregnancy, oligohydramnios and intrauterine growth restriction were detected at week 37, so labor was induced with no complications. Severe thrombocytopenia, neutropenia, increased liver enzymes and acute phase reactants, including ferritin, were detected. On the third day of life, fever and bloody stools appeared. At this point, targeted genetic studies were requested together with supplementary examinations to rule out infectious causes: CSF showed isolated mononuclear lymphocytosis, the microbiological workup (including TORCH infections) was negative. Brain imaging findings are displayed in Fig. 2. In the visual evoked potentials, responses on both sides were not synchronized. At 14 days, Sanger sequencing confirmed he was homozygous carrier of the same pathogenic variant c.2908G > A (p.Ala970Thr) in *ADAR* gene (Fig. 1). Interferon signature was positive in serum using Nanostring nCounter system (6 IRG Z-score = 16.06; 28 IRG Z-score = 11.35) [8, 9]. After ruxolitinib treatment, initiated at 22 days of life (0.2 mg/kg/day, increasing to 0.3 mg/kg/day), fever disappeared, stools became normal and analytic parameters started to improve.

At 2.5 months of age biochemical and hematological parameters normalized, but hypotonia was still present. Ruxolitinib dose was increased to 0.4 mg/kg/day. The patient showed neurological progression with worsening of axial hypotonia, limb hypertonia and pyramidalism, absence of visual tracking, self-limited vertical nystagmus evoked with Moro reflex and irritability. Worsening on brain ultrasound findings was noticed (Fig. 2A). Ruxolitinib dose was increased to 0.7 mg/kg/day, being the treatment well tolerated.

At 4.5 months of life, he was admitted due to febrile community-acquired pneumonia by *Haemophilus influenzae*. After 72 h of appropriate antibiotic treatment, repeated episodes of hypertonia appeared, initially in the upper limbs and later generalized, requiring management in the intensive care unit. These episodes were refractory to treatment, so palliative care was finally established, and the patient deceased at 4 months and 26 days.

## Discussion and conclusions

Aicardi-Goutières Syndrome (AGS) is a monogenic type 1 interferonopathy with infantile onset, characterized by a variable degree of neurological damage. This family illustrates the phenotypic diversity that can arise even in siblings with the same pathogenic variants. Around half of ADAR-related cases have disease presentation after a period of normal development as our patient’s sister (II:4), while our patient (II:5) and presumably his brother (II:1) had symptoms since birth. Regarding the acute symptomatic phase of both siblings (II:4 and II:5), symptoms are compatible with refractory dystonia due to bilateral striatal necrosis [11], a frequently reported phenotype in ADAR-related cases. However, this suspicion could not be confirmed since the necropsy was not authorised.

The genetic variant c.2908G > A occurs at a highly conserved residue in exon 11 and was predicted to be deleterious by in-silico programs. Further, it has been interpreted as a heterozygous variant of uncertain significance in Clinvar and Varsome. GnomAD listed four heterozygous and no homozygous carriers. Exon 11 codes for the catalytic deaminase domain of *ADAR* gene. A defect in this domain could have an important functional effect [12]. Most of the pathogenic missense variants in this location are biallelic. Only two cases with homozygous missense variants were described and one died at 10 months [11]. It was hypothesized that the presence of other deleterious variant in further AGS genes could worsen the clinical outcome, however examination of the rest of AGS genes did not reveal potentially contributing variants [7]. Regarding clinical, radiological and genetic findings in both siblings, the variant c.2908G > A was finally reclassified as likely pathogenic following ACMG criteria.

To our knowledge, this is the youngest patient with AGS-6 treated with JAKI. Given the results obtained with JAKI in other patients described in the literature (Table 1) especially in terms of neurological involvement, as well as the result of the serum interferon signature, which reflects hyperactivation of the interferon pathway in our patient, it was decided to initiate early treatment with JAKI. Ruxolitinib was chosen because it could be prepared in our hospital pharmacy in a dosage form that allowed exact dosing in milligrams according to the patient’s weight. Despite the resolution of the systemic symptoms and normalization of the inflammatory parameters, there was no evidence of neurological improvement.

**Table 1:** Reported use of ruxolitinib in Aicardi-Goutières syndrome (AGS)

Publication (Author-Year)	Diagnose - genetics	Age at treatment	Initial dose	Dose increase-maintenance	Outcome	Comments
Tüngler 2016 [13]	AGS-2 (*RNASEH2B* biallelic variants)	23 months	0,2 mg/kg/day	After 1 week increased to 0,5 mg/kg/day	Reduction of IF signature in 2 weeks. Clinical neurological improvement.	Respiratory infection during treatment without complications, continued treatment. Raised IF during infection, subsequent normalization.
Kothur 2018 [14]	AGS7 (*IFIH1* p. Arg779Cys Heterozygous)	24 months	2,5 mg/12 h	After 6 weeks increased to 5 mg/12 h	Regained stable sitting and walking with walker, improvement in fine manipulation and understanding. MRI: progressive myelination.	Initial low platelets and red blood cells, out of transfusion range
Alsohime 2020 [15] *	Pseudo-TORCH syndrome 2 *(USP18* c.1073 + 1G > A Homozygous)	7 months	5 mg/12 h	After 2 weeks increased to 10 mg/12 h	Sustained improvement: weaning from ventilatory support, healing of necrotizing cellulitis. MRI resolution of hydrocephalus, haemorrhage abd ischemia. At 24 months, Denver II showed developmental age of 9 to 10 months.	At 8 months, reducing the dose to 5 mg/12 h causes clinical worsening. Maintenance dose 10 mg/12 h.
Cattalini 2021 [16]	AGS-6 (*ADAR* c.577 C > G and c.1076_1080 del)	40 months	2,5 mg/12 h	After 10 weeks increased to 5 mg/12 h	Neurological improvement at 18 months. No MRI changes (bilateral pallidal necrosis)	*P. aeruginosa* infection at 9 months of treatment use
Mura 2021 [2]	AGS-2 (*RNASEH2B* c.253 C > G and c.65-13G > A)	18 months	0,4 mg/kg/12 h	0,4 mg/kg/12 h	Sustained improvements on neuromotor and language skills (43 months: sitting without support, 2–3 words sentences). MRI: reduction of T2 white matter hyper intensity, progressive normalization.	Monthly antivaricella IVIG
Li 2022 [17]	AGS-1 (*TREX1* p. G47S and p.C154Mfs*3)	13 years	0,25 mg/kg/day	0,25 mg/kg/day	Febrile attacks and cutaneous lesions subsided. Normalization of ESR, proteinuria and hematuria. Elevation to full score in AGS scale and kept stable. Cognitive improvement. Encephalomalacia on MRI was reduced.	Concomitant use with prednisone, thalidomide and aspirin.
	AGS-7 (*IFIH1* p. A339D Heterozygous)	3 years	0,71 mg/kg/day	0,71 mg/kg/day	Febrile attacks and cutaneous lesions subsided. Catch-up with growth, normalization of head circumference. Elevation to full score in AGS scale and kept stable. Improvement of gross motor and language development. Normalization of ESR.	Concomitant use with prednisone, thalidomide and levothyroxine.
Pararajasingam 2022 [18]	AGS-5 (*SAMHD1* c.427 C > T Homozygous)	5 years	2.5 mg/12 h	After 4 weeks increased to 5 mg/12 h	Generalised panniculitis (post-Covid-19 infection) improved and oedema resolved.	Concomitant use of steroids
Jones 2022 [19]	AGS-6 (*ADAR* c.3019G > A Heterozygous)	2 years	2.5 mg/12 h	2.5 mg/12 h	Improvements in dystonia, vocalisations and social interaction, advancement in motor scores, decrease in neopterin.	Concomitant use of dexamethasone
Individual II.5	AGS-6 (*ADAR* c.2908G > A Homozygous)	22 days	0.2 mg/kg/day	0.7 mg/kg/day	Fever and bloody stools subsided. Liver enzymes and acute phase reactants normalization. Worsening of axial hypotonia, limb hypertonia, absence of visual tracking.	Refractory status dystonicus after community-acquired pneumonia

Abbreviations: IF, interferon; MRI, Magnetic Resonance Imaging; IVIG, Intravenous Immunoglobulin; ESR, Erythrocyte Sedimentation Rate

* Pseudo-TORCH syndrome 2, despite not being AGS was included as it was the youngest patient found on literature with interferonopathy treated with ruxolitinib

To conclude, homozygous variant c.2908G > A (p.Ala970Thr) in *ADAR* gene causes AGS-6. Phenotypic spectrum of the disease varies among individuals with the same pathogenic variant. Early initiation of treatment improved systemic features of AGS but did not prevent the progression of neurological disease.

## Data Availability

All data generated or analyzed during this study are included in this published article.

## Abbreviations

AGS: Aicardi-Goutières Syndrome
CSF: Cerebrospinal fluid
ADAR: Adenosine Deaminase Acting on RNA
JAKIs: Janus Kinase inhibitors
